# A Theory-Based Digital Intervention to Improve Maternal Oral Health Behaviors for Young Children: Quasi-Experimental Study

**DOI:** 10.2196/79002

**Published:** 2026-05-22

**Authors:** Mei Zhao, Min Liu, Shiyu Wang, Xiaoyue Zhang, Chun Chang, Qingping Yun

**Affiliations:** 1 Department of Preventive Dentistry, Beijing Stomatological Hospital, Capital Medical University, Beijing, China; 2 Alice Lee Centre for Nursing Studies, Yong Loo Lin School of Medicine, National University of Singapore, Singapore; 3 Department of Social Medicine and Health Education, School of Public Health, Peking University, Beijing, China; 4 Key Laboratory of Tropical Translational Medicine of Ministry of Education, School of Public Health, Hainan Medical University, NO.3 Xueyuan Road, Longhua district, Haikou, Hainan, China, Haikou, 571199, China, 86 18811182262

**Keywords:** digital intervention, health belief model, oral health, young children, quasi-experimental study

## Abstract

**Background:**

Parental oral health education is critical for preventing early childhood caries. However, few interventions are theoretically grounded or use digital approaches.

**Objective:**

The objective of this study was to evaluate the effects of a health belief model–based digital intervention on maternal oral health behaviors.

**Methods:**

This quasi-experimental study enrolled 648 mother-child dyads from 19 community health care centers (CHCs) in Beijing, China. CHCs were allocated to intervention or control groups depending on their voluntary adoption of the dental referral system. Ten CHCs (n=332, 52.6%) were assigned to the intervention group, where mothers received oral health education materials and had access to a dental referral system. The remaining 9 CHCs (n=316, 47.4%) served as the control group, in which mothers continued to receive standard child health care services. The primary outcome was parent-assisted toothbrushing, and the secondary outcome included other oral health behaviors, including night feeding practices, sugar intake, and dental visits. To evaluate the intervention effects on behavioral outcomes, generalized linear mixed models were used, accounting for repeated measures and potential confounding factors.

**Results:**

Compared with the control group, the intervention group demonstrated a significant increase in parent-assisted toothbrushing, with an absolute difference of 10.3 (95% CI 3.0 to 17.6; *P*=.006) percentage points at 6 months and 1.5 (95% CI −7.2 to 10.1; *P*=.74) percentage points at 12 months. Additionally, dental visit rates were significantly higher in the intervention group at 12 months (odds ratio 4.65, 95% CI 1.30 to 16.70; *P*=.02). However, no statistically significant differences were observed between groups in nighttime feeding cessation or sugar intake control at either the 6- or 12-month follow-ups.

**Conclusions:**

The health belief model–based digital intervention was effective in the short term for enhancing parent-assisted toothbrushing in young children, but its long-term effectiveness remains unproven. Future research should therefore prioritize exploring sustainability strategies.

## Introduction

### Background

Early childhood caries (ECC) is one of the most prevalent chronic conditions affecting children worldwide [1]. The Global Burden of Disease Study (2017) estimated approximately 532 million cases of untreated caries in primary teeth [2], and the epidemiological data from 193 countries (2007‐2017) revealed that 57.3% of children aged 3 to 6 years were affected by dental caries [3]. In China, the situation appears even more concerning, with the national oral health survey (2015) reporting that 71.9% of children aged 5 years were affected by ECC [4]. Beyond its immediate detrimental effects on young children’s quality of life, ECC significantly elevates the risk of caries in permanent dentition, resulting in long-term oral health consequences [5,6].

Parents play an important role in their young children’s oral health care. Key recommended practices for infants’ and toddlers’ oral care include parent-assisted toothbrushing, limiting dietary sugar intake, avoiding prolonged nighttime feeding, and scheduling regular dental visits [7]. Despite the importance of these practices, most parents fail to adhere to the recommended oral health behaviors [8,9].

Although numerous oral health education programs have been designed for parents, their efficacy across different studies remains mixed [10]. A critical issue is that most interventions lacked theoretical frameworks, limiting their ability to explain intervention outcomes [10]. Moreover, existing programs predominantly emphasize home-based oral care behaviors while neglecting the persistently low rates of dental visits among young children [11,12]. This oversight is problematic because early dental visits enable oral health professionals to deliver personalized oral health education and help families adopt optimal oral health practices [13]. Nevertheless, strategies to effectively promote dental visit adherence remain poorly investigated.

Interventions grounded in theoretical frameworks tend to be more effective, underscoring the value of theory-driven approaches in health behavior change [14]. The health belief model (HBM) is a well-validated social psychological framework for health behavior modification [15]. The model’s core constructs, including perceived susceptibility, perceived severity, perceived benefits, perceived barriers, and self-efficacy, offer a comprehensive theoretical framework for intervention design. A previous review has demonstrated its efficacy in improving oral health behaviors among adolescent and adult populations [16], yet its application in parental oral health education programs remains unexplored. To date, only one relevant study protocol has been published [17]. That study delivered HBM-based SMS text messaging interventions to parents of children aged 18 to 30 months, but it focused only on toothbrushing and sugar intake, without addressing early dental visits. Moreover, an existing intervention used caries prevalence and complication statistics to increase parents’ awareness of ECC susceptibility and severity, but it did not address other important factors, such as perceived benefits and barriers to performing recommended oral health behaviors [18]. Therefore, the effectiveness of comprehensive HBM-based interventions in promoting early childhood oral health behaviors remains to be investigated.

### Aims

To address this gap, we developed a novel digital intervention grounded in the HBM. To improve the low rate of dental visits among young children, the intervention integrated a dental referral system to facilitate access to dental care by helping parents locate nearby clinics and book appointments directly. The primary objective of this study was to evaluate the effects of this HBM-based digital intervention on improving maternal oral health behaviors for young children.

## Methods

### Design, Participants, and Recruitment

A parallel, quasi-experimental design was used to evaluate the effects of the HBM-based digital intervention on maternal oral health behaviors for young children aged 11 to 14 months. Participants were eligible if they met the following criteria: (1) aged 11 to 14 months, (2) at least one tooth has erupted, and (3) no cleft lip or palate. Children diagnosed by a physician with a chronic disease were excluded. The potential participants were recruited from 19 community health care centers (CHCs) in Beijing, China, between September and November 2020. Mothers of infants aged 11 to 12 months were approached during routine visits to community vaccination clinics, while those with children aged 13 to 14 months were contacted via telephone based on vaccination registry records and were invited to participate in this study. Mothers who agreed to participate were then asked to review and sign the informed consent form at the CHC.

CHCs were assigned to the intervention or control group based on their voluntary decision to implement the dental referral system with an allocation ratio of 1:1. Of the 19 CHCs, 10 (52.6%) that opted to implement the system formed the intervention group, while the remaining 9 (47.4%) served as the control group. To mitigate potential selection bias between treatment groups, we implemented controls for a set of family- and child-related factors known to be associated with young children’s oral health in the literature [19-21] (refer to the Control Variables section).

### Intervention and Control Group

**Figure 1.**
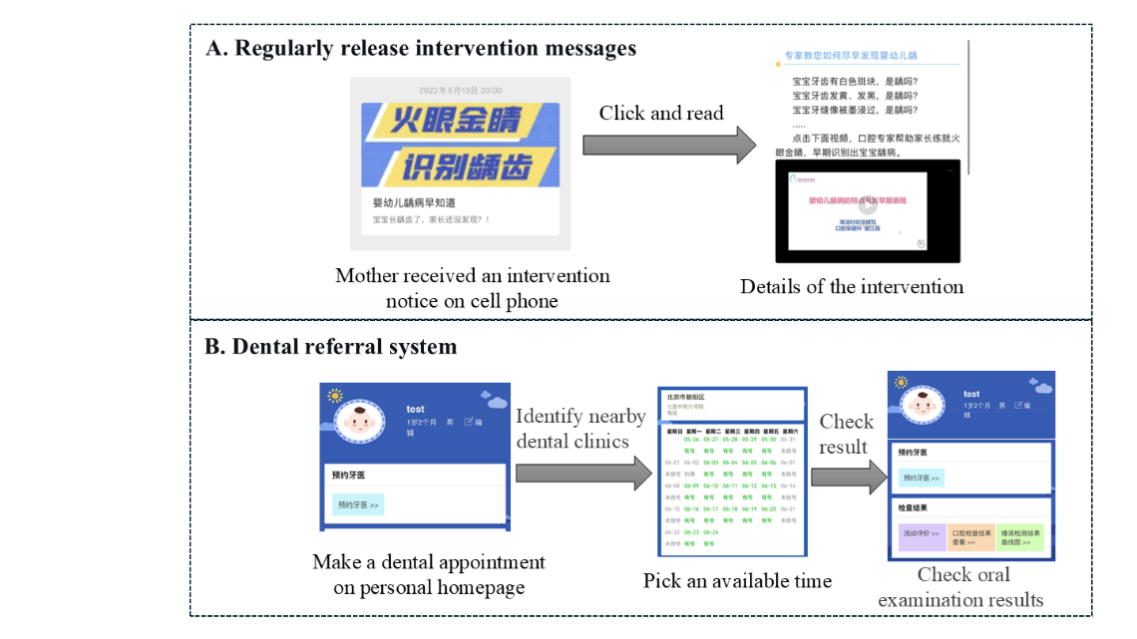
Key elements of the intervention program. (A) Regular intervention message to parents; (B): dental referral system for appointment booking.

Mothers in the intervention group received the intervention through a WeChat (Tencent Holdings Limited) public account, via which we disseminated the intervention materials and hosted the dental referral system (Figure 1). The intervention lasted for 6 months, divided into two phases: (1) an intensive phase (first 2 months) with weekly intervention material deliveries, followed by (2) a maintenance phase (remaining 4 months) with biweekly material distribution. Throughout the intervention period, participants could use the online dental referral system to book dental appointments for their children. Guided by HBM, the intervention materials targeted the following constructs: (1) perceived susceptibility—presented Chinese epidemiological data on high prevalence of ECC [4]; (2) perceived severity—emphasized the adverse developmental consequences of untreated ECC; (3) perceived benefits—outlined advantages of oral health behaviors, including impacts on children’s dental health, speech development, and long-term oral health outcomes [22,23]; (4) perceived barriers—provided practical strategies to address common obstacles (eg, managing child resistance during toothbrushing) [24]; and (5) self-efficacy—built confidence through the sharing of peer maternal experiences and success stories [25]. All content was reviewed and approved by a panel of 8 experts (including 3 oral health professionals, 2 pediatricians, and 2 health promotion specialists) to ensure scientific accuracy and appropriateness. Furthermore, the digital platform’s usability and accessibility were evaluated in a 2-phase process: an initial assessment by the research team, followed by a test with 8 mothers for user-friendliness and clarity. Feedback from these processes was incorporated prior to the final release. The detailed intervention materials, including the topic, key contents, corresponding theoretical constructs, and engagement metrics (eg, views) for each session, are provided in Multimedia Appendix 1.

Mothers in the control group received standard child health care as stipulated by the national health management service for children aged 0 to 6 years [26], which for 1- to 2-year-olds includes biannual general physical examinations and feeding guidance. We did not provide any additional interventions to the control group.

### Outcomes and Measurements

Due to COVID-19 pandemic–related restrictions, this study modified its primary outcome from oral health assessments to parent-assisted toothbrushing behaviors. This adaptation was made for two reasons: (1) standardized caries detection became impossible during the epidemic and (2) as a key ECC-preventive behavior [27], toothbrushing demands daily practice, reflecting ongoing parental commitment to their child’s oral care. Other oral health behaviors, including nighttime feeding cessation, dental visits, and sugar intake control, served as secondary outcomes in this study. Consistent with our theoretical framework, the intervention aimed to improve maternal oral health behaviors by knowledge enhancement and psychosocial modification, with these factors concurrently assessed as secondary outcome measures.

All data were obtained using structured questionnaires at CHCs. Parent-assisted toothbrushing was operationally defined as the daily toothbrushing performed by caregivers before bedtime. Accordingly, the frequency of toothbrushing and whether it occurred before bedtime were recorded. The use of fluoridated toothpaste was also documented. Following biannual dental visit recommendations for young children, we assessed whether they had received dental visits in the preceding 6 months. Regarding nighttime feeding practices, mothers were asked whether they had discontinued nighttime feeding for their young children; responses indicating “yes” were categorized as nighttime feeding cessation. Additionally, data on the frequency of sugar intake, including candies, sugar-sweetened beverages, and bakery products, were collected.

Maternal oral health knowledge was evaluated using a 9-item questionnaire, which was developed according to Chinese infants’ and toddlers’ oral health guidelines (Multimedia Appendix 2) [23]. Each item offered 3 response options: “true,” “false,” and “don't know.” Correct answers received 1 point, while incorrect or “don’t know” responses scored 0.

This study also assessed HBM components, including perceived susceptibility to ECC, perceived severity of ECC, perceived benefits and barriers of oral health behaviors, and self-efficacy. All components were measured using 5-point Likert scale items (1=strongly disagree to 5=strongly agree), with cumulative scores calculated for each domain. A pilot study was conducted with 187 mothers (excluded from the main study) to assess the scales’ readability and internal consistency. These scales demonstrated acceptable internal consistency, with Cronbach α coefficients ranging from 0.67 to 0.93. The items for each HBM component are presented in Multimedia Appendix 3.

### Blinding

Blinding of participants was not possible due to the nature of this quasi-experimental study. Furthermore, as the primary outcome was self-reported, blinding of the outcome assessors was not applicable.

### Sample Size

On the basis of our previous survey in Beijing, the rate of parent-assisted toothbrushing among 2-year-old children was 22.6%. This rate served as the reference for the control group, while the expected rate in the intervention group was set at 33% (ie, the prevention fraction of 50% based on a previous study [28]). The minimum necessary sample size was 258 participants for each arm based on a 2-sided test at the .05 level of significance and 80% power. Assuming a 20% loss of participants to follow-up, 310 participants were needed in each arm.

### Control Variables

This study included 7 covariates encompassing both child and maternal characteristics. Child-related covariates included sex (male vs female), baseline age (categorized as 11‐12 months vs 13‐14 months), order of birth (first child vs nonfirst child) and primary caregiver status (mother vs others). Maternal characteristics included infant feeding practices, educational attainment, and household income. Infant feeding practices (0‐6 months) were categorized into 3 groups: exclusive breastfeeding, mixed feeding, or exclusive formula feeding. Educational attainment was classified into 3 levels: college and below, bachelor’s degree, or graduate degree. Based on participants’ self-ranking of their family income relative to others in the local area, household income was classified into 3 tiers, including high (top 40%), middle (60%‐80%), or low (bottom 40%). The selection of birth order and infant feeding practices as covariates is supported by previous evidence linking them to children’s oral health outcomes [19,20].

### Follow-Up

Consistent with World Health Organization recommendations for evaluating novel digital health interventions, this study incorporated medium- and long-term impact assessments through 3-wave data collection [29]. Thus, data collection occurred at 3 time points in this study: outcome variables were assessed at baseline, 6 months (immediately after the intervention), and 12 months (6 months after the postintervention assessment). Maternal oral health knowledge and psychosocial variables were measured at baseline and 12 months, and control variables were obtained at baseline.

### Statistical Analysis

Behavioral outcomes and control variables were reported as frequencies (proportions) and analyzed by chi-square test or Fisher exact test, as appropriate. Maternal oral health knowledge and psychosocial variables were described as mean (SD) and compared using 2-tailed independent *t* tests. Logistic regression generalized linear mixed models were used to assess behavioral outcome effects, and linear mixed models were applied to assess the psychosocial outcome effects. Fixed effects included time, treatment group, and their interaction (time×treatment). Random effects included community, accounting for within-cluster correlations. A statistically significant interaction indicates that the treatment effect varied over time.

Data analysis followed the intention-to-treat principles. Participants who had at least one follow-up visit were included in the primary analysis. Missing data were accounted for by a mixed-effects model using the full information maximum likelihood estimation method [30]. Sensitivity analyses were conducted to evaluate the robustness of the primary results. First, a complete-case analysis was performed. Second, given the continuous nature of the behavior, the last observation carried forward (LOCF) approach was applied under the assumption that the behavior would remain unchanged after the last measurement. Analyses were conducted using R software (version 4.4; R Foundation for Statistical Computing).

### Ethical Considerations

Ethics approval (IRB00001052-19136) was obtained from the institutional review board of Peking University Health Science Center. This study was performed in accordance with the 1964 Declaration of Helsinki and its later amendments or comparable ethical standards. Informed consent was obtained from all mothers. All data were anonymized prior to analysis. No personally identifiable information was included in the analysis or the reporting of findings. Participants received a pack of baby wipes (valued at RMB 50 [US $7.30]) on each visit. No additional financial compensation was offered for participation.

## Results

### Baseline Characteristics

**Figure 2.**
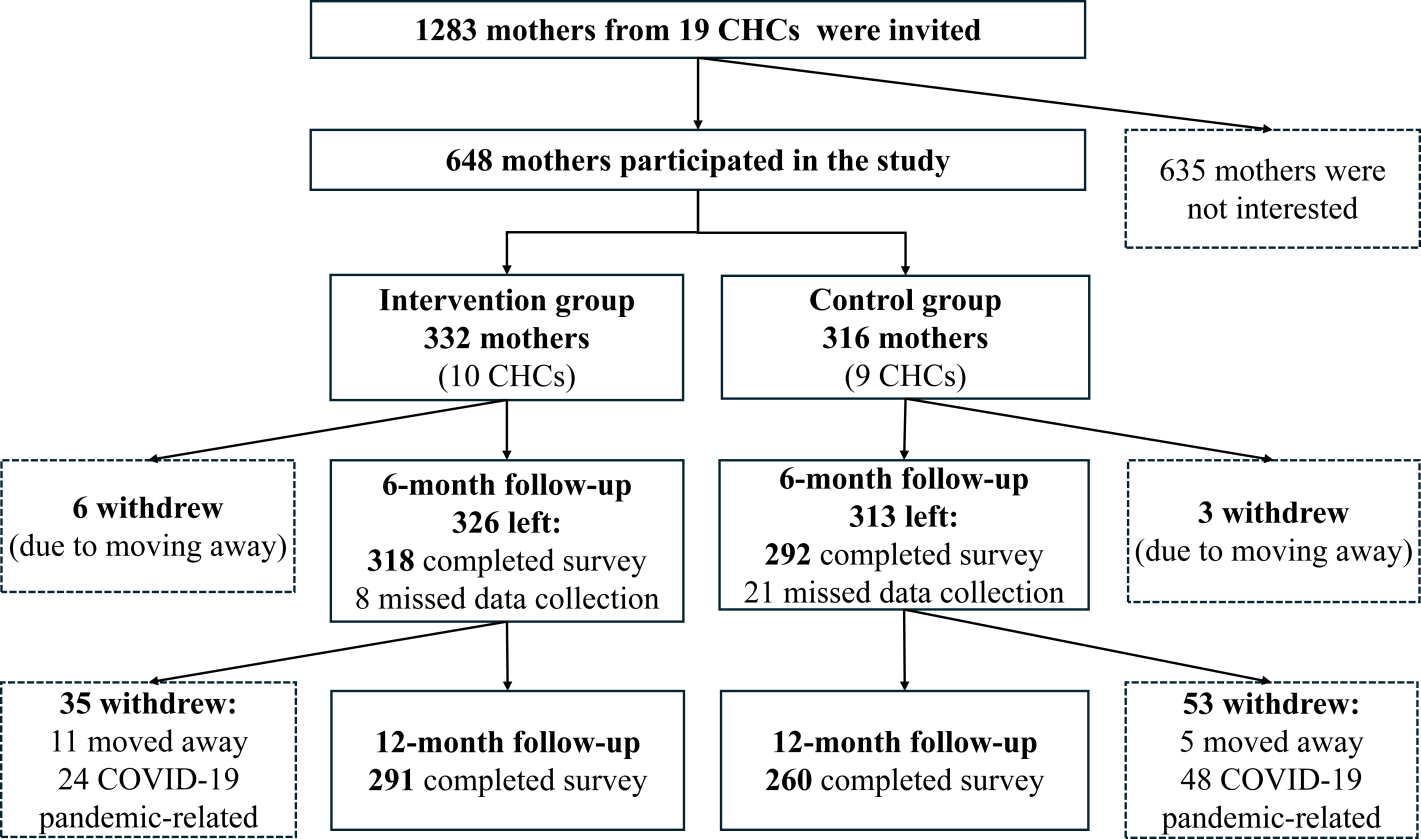
Flow diagram of participants. CHC: community health care center.

This study enrolled 648 mother-child dyads. Of the children, 331 (51.1%) were male, and 546 (84.3%) were aged 11 to 12 months. Maternal education levels were distributed as follows (Table 1): 508 (78.4%) held at least a bachelor’s degree, 103 (15.9%) had a college degree, and 37 (5.7%) completed high school or less. The baseline characteristics of participants were well matched between the intervention and control groups. Follow-up rates were 94.1% (n=610) at 6 months and 85% (n=551) at 12 months, meeting prespecified expectations (Figure 2).

**Table 1.** Baseline characteristics of participants.

Characteristics	Total (n=648), n (%)	Intervention group (n=332), n (%)	Control group (n=316), n (%)	*P* value
Child sex				.80
Male	331 (51.1)	168 (50.6)	163 (51.6)	
Female	317 (48.9)	164 (49.4)	153 (48.4)	
Child age group (months)				.63
11‐12	546 (84.3)	282 (84.9)	264 (83.5)	
13‐14	102 (15.7)	50 (15.1)	52 (16.5)	
Birth order				.36
First child	462 (71.3)	242 (72.9)	220 (69.6)	
Non–first child	186 (28.7)	90 (27.1)	96 (30.4)	
Primary caregiver				.80
Mother	294 (45.4)	149 (44.9)	145 (45.9)	
Others	354 (54.6)	183 (55.1)	171 (54.1)	
Infant feeding practice				.76
Exclusive breastfeeding	339 (52.3)	175 (52.7)	164 (51.9)	
Mixed feeding	224 (34.6)	111 (33.4)	113 (35.8)	
Exclusive formula feeding	85 (13.1)	46 (13.9)	39 (12.3)	
Maternal education				.15
High school or below	37 (5.7)	16 (4.8)	21 (6.6)	
College (2‐3 y)	103 (15.9)	61 (18.4)	42 (13.3)	
Bachelor’s degree or higher	508 (78.4)	255 (76.8)	253 (80.1)	
Household income				.30
High	157 (24.2)	75 (22.6)	82 (25.9)	
Middle	459 (70.8)	237 (71.4)	222 (70.3)	
Low	32 (5)	20 (6)	12 (3.8)	

Of the initial sample, 38 (5.9%) participants did not complete the 6-month follow-up. Compared with completers, noncompleters had significantly higher baseline self-efficacy scores (*P*=.03) and a higher prevalence of dental visits (*P*=.003) at baseline (Multimedia Appendix 4).

### Behavioral Outcome

The intervention demonstrated significant short-term effects on parent-assisted toothbrushing behavior. Compared with controls, mothers in the intervention group showed a 10.3 percentage point increase (95% CI 3.0-17.6) in toothbrushing behavior, corresponding to an adjusted odds ratio (OR) of 2.43 (95% CI 1.24-4.74). The observed improvement was consistent in sensitivity analyses using complete-case analysis (adjusted OR 2.19, 95% CI 1.06-4.53; Multimedia Appendix 5) and the LOCF approach to handle missing data (adjusted OR 2.88, 95% CI 1.43-5.78; Multimedia Appendix 6). However, this improvement was not sustained at the 12-month assessment (Table 2), with an adjusted OR of 1.20 (95% CI 0.61-2.36). No statistically significant effect was found for toothbrushing with fluoridated toothpaste at this follow-up as well (Multimedia Appendix 7), with an adjusted OR of 2.12 (95% CI 0.78-5.75).

**Table 2.** Intervention effects on maternal oral health behaviors for young children.

	Intervention group, n/N (%)	Control group, n/N (%)	Rate difference, % (95% CI)	*P* value	Odds ratio (95% CI)	*P* value
Parent-assisted brushing						
Baseline	47/332 (14.2)	47/316 (14.9)	−0.3 (−2.5 to 1.7)	.74	1.00^a^	—^b^
6-month follow-up	124/318 (39)	82/292 (28.1)	10.3 (3.0 to 17.6)	.006	2.43 (1.24 to 4.74)	.009
12-month follow-up	118/291 (40.5)	104/260 (40)	1.5 (−7.2 to 10.1)	.74	1.20 (0.61 to 2.36)	.59
Nighttime feeding cessation						
Baseline	104/332 (31.3)	79/316 (25)	1.0 (−4.5 to 6.5)	.73	1.00	—
6-month follow-up	225/318 (70.8)	214/292 (73.3)	−0.6 (−3.3 to 2.0)	.64	0.53 (0.16 to 1.73)	.29
12-month follow-up	253/291 (86.9)	222/260 (85.4)	0.0 (−0.1 to 0.2)	.67	1.21 (0.23 to 6.32)	.82
Sugar intake control						
Baseline	259/332 (78)	241/316 (76.3)	2.4 (−4.8 to 9.8)	.51	1.00	—
6-month follow-up	220/318 (69.2)	212/292 (72.6)	−3.5 (−12.2 to 9.2)	.43	0.73 (0.41 to 1.28)	.27
12-month follow-up	190/291 (65.3)	172/260 (66.2)	0.1 (−9.8 to 10.0)	.99	0.87 (0.49 to 1.53)	.62
Dental visit in past 6 months						
Baseline	9/332 (2.7)	9/316 (2.8)	−0.1 (−0.7 to 0.5)	.65	1.00	—
6-month follow-up	33/318 (10.4)	15/292 (5.1)	1.5 (−0.1 to 3.6)	.15	3.29 (0.98 to 11.05)	.05
12-month follow-up	30/291 (10.3)	13/260 (5)	2.0 ( –0.5 to 4.4)	.12	4.65 (1.30 to 16.70)	.02

a Odds ratio value of 1.00 indicates the reference group in the model.

b Not applicable.

Additionally, the intervention showed a limited effect on dental visits. Mothers in the intervention group had a higher likelihood of taking their children for dental visits compared with controls, with adjusted ORs of 3.29 (95% CI 0.98‐11.05) at 6 months and 4.65 (95% CI 1.30‐16.70) at 12 months. However, the low incidence of dental visits led to wide CIs in both the primary and sensitivity analyses. The sensitivity analyses yielded ORs of 1.81 (95% CI 0.29-11.38) for the complete-case analysis (Multimedia Appendix 5) and 3.85 (95% CI 0.82-17.95) for the LOCF approach at 12-month follow-up (Multimedia Appendix 6). No statistically significant effects of the intervention were observed on either nighttime feeding cessation or sugar intake reduction at the 6- or 12-month follow-ups (*P*>.05).

### Maternal Knowledge and Psychosocial Outcomes

We further assessed changes in maternal knowledge and psychosocial variables over the 12-month study period. The intervention significantly improved maternal oral health knowledge scores (mean difference 0.31 points; 95% CI 0.01-0.60), whereas no statistically significant changes were observed in other psychosocial components (Table 3).

**Table 3.** Intervention effects on maternal oral health knowledge and health belief model constructs.

	Intervention group, mean (SD)	Control group, mean (SD)	Mean difference (95% CI)	*P* value
Maternal oral health knowledge				
Baseline	6.24 (1.56)	6.25 (1.75)	−0.03 (−0.31 to 0.25)	.82
12-month follow-up	7.15 (1.43)	6.90 (1.51)	0.31 (0.01 to 0.60)	.04
Perceived susceptibility to ECC^a^				
Baseline	11.66 (1.72)	11.73 (1.89)	−0.08 (−0.44 to 0.27)	.65
12-month follow-up	12.11 (1.82)	11.91 (2.01)	0.21 (−0.17 to 0.59)	.28
Perceived severity of ECC				
Baseline	14.28 (1.50)	14.22 (1.70)	0.05 (−0.23 to 0.33)	.72
12-month follow-up	14.46 (1.27)	14.28 (1.64)	0.19 (−0.11 to 0.48)	.23
Perceived benefits of behaviors				
Baseline	28.89 (2.75)	28.73 (2.94)	0.15 (−0.31 to 0.60)	.53
12-month follow-up	29.40 (1.72)	29.02 (3.01)	0.38 (−0.11 to 0.86)	.14
Perceived barriers of behaviors				
Baseline	22.02 (6.13)	22.00 (6.58)	−0.03 (−1.16 to 1.10)	.96
12-month follow-up	20.82 (5.46)	20.75 (6.04)	0.19 (−1.00 to 1.37)	.76
Self-efficacy				
Baseline	13.56 (1.79)	13.72 (1.95)	−0.15 (−0.48 to 0.19)	.40
12-month follow-up	13.56 (1.78)	13.45 (2.01)	0.17 (−0.18 to 0.52)	.34

a ECC: early childhood caries.

## Discussion

### Main Findings

In this quasi-experimental study, the HBM-based digital intervention achieved a 10.3 percentage point improvement in parent-assisted toothbrushing behavior at the 6-month follow-up, but this improvement did not persist to the 12-month follow-up. Additionally, slight improvements were observed in dental visit adherence and maternal oral health knowledge at the 12-month follow-up.

### Comparison With Prior Work

Previous interventions targeting mothers of young children (aged 0-3 years) primarily focused on oral health knowledge and skills without solid theoretical foundation [18,31,32]. Among the few theory-guided interventions, one used the theory of planned behavior (TPB). While this intervention improved maternal attitudes and perceived behavioral control, it did not yield significant changes in behavioral intention or toothbrushing behavior at the 3-month follow-up [28]. This limited efficacy may be attributed to 2 key conceptual omissions in TPB. First, TPB does not incorporate self-efficacy, which is crucial for sustaining parental involvement in children’s oral hygiene [33]. Second, it overlooks the perceived threat, a construct whose enhancement has been linked to reduced caries incidence in children [18]. HBM integrates these essential components. Consequently, it was selected as the theoretical framework for this study.

Guided by HBM, our study achieved short-term improvements in toothbrushing behavior, likely mediated by increased maternal oral health knowledge [34], as our results indicated that maternal oral health knowledge scores increased 0.31 points from baseline. However, the intervention effects diminished by the 12-month follow-up, possibly due to persistent perceived barriers or inadequate self-efficacy reinforcement [24,35]. To facilitate lasting behavioral modifications, future research should explore the efficacy of hybrid interventions that integrate initial education with booster sessions (eg, regular reminders) to address behavioral relapses. Future practice should embed oral health promotion into routine child health services (eg, during vaccination visits), thereby creating systematic opportunities for timely problem-solving to overcome barriers and for skill-refreshing to enhance self-efficacy.

Previous interventions focused on home-based oral behaviors and largely overlooked the importance of dental visits [10,36]. To our knowledge, this study is the first to implement an online dental referral system designed to facilitate parental scheduling of dental appointments for children. However, the intervention yielded only a modest effect, with a mere 2.0 percentage point increase in dental visit rates at the 12-month follow-up. This limited effect is likely multifactorial. First, the COVID-19 pandemic created a universal barrier, during which families avoided nonessential pediatric dental care [37]. More fundamentally, our digital dental referral system primarily addressed the barriers of finding and scheduling an appointment, but it could not overcome other critical obstacles, such as financial constraints [38], and parental attitudes regarding dental visits. If mothers primarily view dental visits as therapeutic rather than a preventive service, they are unlikely to take their children to oral health professionals in the absence of symptoms [39,40]. This suggests that future interventions need to address not only service access but also cost barriers and underlying beliefs about dental visits.

It should be noted that this study was likely underpowered to detect a significant effect on dental visits, with the low incidence of this behavior resulting in wide CIs. This limits the robustness of our findings and calls for cautious interpretation of the results. Therefore, future studies with larger sample sizes are required to evaluate the intervention’s effect.

However, our intervention showed no significant effect on nighttime feeding cessation. Further analysis revealed that the mean cessation age was similar between groups (intervention: mean 13.48, SD 4.18 months vs control: mean 13.41, SD 3.86 months; *P*=.79). This overall cessation timing closely aligned with the baseline age of participants (11‐14 months), which could partially explain the limited intervention effect.

### Strengths and Limitations

This theory-based digital intervention evaluated both oral health behavior changes and shifts in maternal psychosocial factors, offering explanatory insights into the mechanisms behind behavioral improvements. However, the study had several limitations. First, randomization was not feasible due to COVID-19 pandemic–related constraints that prevented comprehensive implementation of the referral intervention across participating CHCs. The lack of randomization limited our ability to fully account for potential confounders [41]. To mitigate this, we used a parallel group comparison and adjusted for child- and maternal-related confounding variables, strengthening causal inference. Second, the COVID-19 pandemic prevented clinical assessments of oral health outcomes (eg, caries incidence), restricting our analysis to rely on self-reported behaviors, which might introduce reporting bias. Future studies can more comprehensively assess the effect of theory-based digital interventions on children’s oral health.

### Conclusions

The HBM-based digital intervention was effective in the short term for enhancing parent-assisted toothbrushing in young children, but its long-term effectiveness remains unproven. Future research should therefore prioritize exploring sustainability strategies.

## Supplementary material

10.2196/79002 Multimedia Appendix 1 Topics and key contents of intervention materials.

10.2196/79002 Multimedia Appendix 2 Maternal oral health knowledge questionnaire.

10.2196/79002 Multimedia Appendix 3 Items measuring health belief model constructs.

10.2196/79002 Multimedia Appendix 4 Baseline characteristics of participants by follow‐up completion.

10.2196/79002 Multimedia Appendix 5 Intervention effects on maternal oral health behaviors: Based on complete-case analysis.

10.2196/79002 Multimedia Appendix 6 Intervention effects on behavioral outcomes: Based on the last observation carried forward approach.

10.2196/79002 Multimedia Appendix 7 Intervention effects on toothbrushing with fluoridated toothpaste.

10.2196/79002 Checklist 1 CONSORT-eHealth (V 1.6.1).

## Abbreviations

CHC: community health care center
ECC: early childhood caries
HBM: health belief model
LOCF: last observation carried forward
OR: odds ratio
TPB: theory of planned behavior
