# Supplementary material for: A Theory-Based Digital Intervention to Improve Maternal Oral Health Behaviors for Young Children: Quasi-Experimental Study
Source: JMIR Mhealth Uhealth. 2026 May 22;14:e79002. doi: 10.2196/79002 (PMC13197111; doi:10.2196/79002)
Supplement: Multimedia Appendix 1 [file mhealth-v14-e79002-s001.docx]

| **Multimedia appendix 1.** **Intervention Curriculum: Topics, content, and corresponding health belief model (HBM) constructs** | | | |
| --- | --- | --- | --- |
| **Session** | **Topic** | **Key content (HBM components)** | Views  (Reach rate) |
| 1 | General introduction of children tooth eruption and oral care | - Illustrate the critical role of oral care in children's dental development (Perceived benefits) - Empower mothers to initiate oral care early (Self-efficacy) | 254  (76.5%) |
| 2 | Epidemiological profile and health consequences of early childhood caries (ECC) | - Highlight the high prevalence of ECC among young children in China (Perceived susceptibility) - Outline severe consequences: pain, sleep and eating difficulties, growth impairment, and long-term oral health issues (Perceived severity) | 165  (49.7%) |
| 3 | ECC pathogenesis and night feeding cessation | - Explain how prolonged night feeding contributes to ECC development (Perceived susceptibility/severity) - Introduce the recommended weaning window - Provide guidance on gradually reducing night feeding (Perceived benefits/barriers) | 189  (56.3%) |
| 4 | Video tutorial on parental-assisted toothbrushing for children | - Demonstrate proper techniques and explain key steps (Perceived susceptibility/severity) - Encourage parents to begin brushing early (Self-efficacy) | 316  (95.5%) |
| 5 | How to choose toothbrushes and toothpaste for children | - Core Principles:   Ensure safety and effectiveness (Perceived benefits)  Involve children in selection (Perceived barriers) | 95  (28.6%) |
| 6 | Video tutorial on parental-assisted flossing for children | - Demonstrate correct flossing techniques and explain their importance (Perceived benefits/barriers) | 106  (31.9%) |
| 7 | First dental visit (FDV): Procedure and clinical significance | - Explain the timing and importance of FDV as a preventive milestone, not just a problem-based checkup (Perceived benefits) - Remind parents to utilize the provided dental referral system (Perceived barriers) | 116  (34.9%) |
| 8 | Transitioning from bottles/breastfeeding to open-cup drinking | - Explain why transition is crucial: prevents ECC and supports oral motor development (Perceived benefits) - Guide parents on how to choose an appropriate cup (Perceived barriers) | 101  (30.4%) |
| 9 | Oral development issues in infants and young children | - Identify common oral development issues and their link to ECC - Highlight detrimental oral habits and their association with ECC (Perceived Susceptibility/Severity) | 99  (29.8%) |
| 10 | Maternal experience in night feeding cessation | - Share real-life maternal experiences on successful cessation:   Challenges encountered and family support strategies (Perceived barriers)   - Positive changes observed after cessation (Perceived benefits, Self-efficacy) | 273  (82.2%) |
| 11 | Tips for brushing children's teeth | - Share practical maternal experiences on successful brushing: Common challenges and effective coping strategies (Perceived Benefits/Barriers, Self-efficacy) | 151  (45.5%) |
| 12 | Purchasing guide for toddler oral hygiene product | - Core Principles:   Ensure safety and effectiveness (Perceived benefits)  Involve children in selection (Perceived barriers) | 135  (40.7%) |
| 13 | Debunking dental fatalism | - Emphasize that behavioral factors (diet and hygiene) are the primary drivers of tooth decay, not fate (Self-efficacy) | 59  (17.8%) |
| 14 | Early clinical manifestations of ECC | - Present the early signs and symptoms of ECC (Perceived susceptibility/severity) | 86  (25.9%) |
| 15 | Fluoride varnish: Procedure and clinical significance | - Explain the importance and benefits of fluoride varnish (Perceived benefits) - Remind parents to utilize the provided dental referral system (Perceived barriers) | 54  (16.3%) |
| 16 | Expert interpretation of the ECC prevention guidelines | - Emphasize that daily care is an effective strategy for preventing ECC (Perceived benefits, self-efficacy) | 44  (13.3%) |
