# Supplementary material for: A Theory-Based Digital Intervention to Improve Maternal Oral Health Behaviors for Young Children: Quasi-Experimental Study
Source: JMIR Mhealth Uhealth. 2026 May 22;14:e79002. doi: 10.2196/79002 (PMC13197111; doi:10.2196/79002)
Supplement: Multimedia Appendix 2 [file mhealth-v14-e79002-s002.docx]

| **Multimedia appendix 2. Maternal oral health knowledge questionnaire** | | | |  |
| --- | --- | --- | --- | --- |
| Items | Options | | |  |
|  | True | False | Don't know | |
| (1) Tooth decay occurs when bacteria use sugars to make acids that attack and damage teeth. |  |  |  | |
| (2) Prolonged nighttime feeding with milk/formula may cause caries. |  |  |  | |
| (3) Oral hygiene should begin from birth. |  |  |  | |
| (4) Frequently adding calcium tablets, syrup, or concentrated juice to child’s bottle can raise their risk of tooth decay. |  |  |  | |
| (5) Children younger than 3 years old cannot use fluoride toothpaste. |  |  |  | |
| (6) Children younger than 3 years old cannot use dental floss. |  |  |  | |
| (7) Frequent between-meal snacking may cause caries. |  |  |  | |
| (8) The child's teeth just erupted and are relatively healthy, so there is no need for an oral examination. |  |  |  | |
| (9) Permanent teeth could replace primary teeth, so if they are broken, they do not need treatment. |  |  |  | |
