# Supplementary material for: A Theory-Based Digital Intervention to Improve Maternal Oral Health Behaviors for Young Children: Quasi-Experimental Study
Source: JMIR Mhealth Uhealth. 2026 May 22;14:e79002. doi: 10.2196/79002 (PMC13197111; doi:10.2196/79002)
Supplement: Multimedia Appendix 3 [file mhealth-v14-e79002-s003.docx]

| Multimedia appendix 3. Items measuring health belief model constructs. | | |
| --- | --- | --- |
| Constructs | Items ^a^ | Scoring range ^b^ |
| Perceived susceptibility to early childhood caries (ECC) | (1) Many children in my community suffer from caries. | 3‐15 |
|  | (2) My child is more prone to caries than other children. |  |
|  | (3) If I do not assist my child with brushing their teeth, they are likely to develop caries. |  |
| Perceived severity to ECC | (1) Caries can impact a child's eating and growth development. | 3-15 |
|  | (2) Children may experience pain if they have caries. |  |
|  | (3) Caries can affect a child's overall health. |  |
| Perceived benefits of behavior | (1) Avoiding prolonged night feeding reduces risk of caries. | 6-30 |
|  | (2) Avoiding saliva contact with children helps reduce the spread of harmful oral bacteria from adults. |  |
|  | (3) Brushing my child's teeth can help them prevent caries. |  |
|  | (4) Brushing my child’s teeth makes their teeth looks good. |  |
|  | (5) Regular dental visits help to prevent the onset of oral diseases. |  |
|  | (6) Timely treatment is helpful to oral health when children have caries. |  |
| Perceived barriers of behavior | (1) Discontinuing night feeding will make my baby cry. | 7-35 |
|  | (2) I don’t know how to brush my child’s teeth. |  |
|  | (3) Brushing my child's teeth takes up some of my time. |  |
|  | (4) My child doesn't cooperate when I brush their teeth. |  |
|  | (5) I do not know where to access paediatric oral health services. |  |
|  | (6) I do not have enough time to take my child to a dentist. |  |
|  | (7) Taking children to a dentist will cost a lot of money. |  |
| Self-efficacy | 1. I can make the best effort to keep my baby free from caries. | 3-15 |
|  | 1. Even though my child doesn't cooperate, I can still manage to brush their teeth. |  |
|  | 1. I feel confident in my ability to maintain my child's regular dental visits despite a busy schedule. |  |
| a: Items were rated on a 5-point Likert scale from 1 (strongly disagree) to 5 (strongly agree).  b: The scoring range for each construct represents the possible total score, calculated as the sum of the lowest and highest possible scores from all items within that construct. | | |
