# Supplementary material for: A Theory-Based Digital Intervention to Improve Maternal Oral Health Behaviors for Young Children: Quasi-Experimental Study
Source: JMIR Mhealth Uhealth. 2026 May 22;14:e79002. doi: 10.2196/79002 (PMC13197111; doi:10.2196/79002)
Supplement: Multimedia Appendix 4 [file mhealth-v14-e79002-s004.docx]

| **Multimedia appendix 4. Baseline characteristics of participants by follow‐up completion** | | | |
| --- | --- | --- | --- |
|  | Complete 6-month follow up | Incomplete 6-month follow up | *P* value |
| **Study group** |  |  | .067 |
| Intervention | 318 (95.8%) | 14 (4.2%) |  |
| Control | 292 (92.4%) | 24 (7.6%) |  |
| **Child gender** |  |  | .595 |
| Male | 310 (93.7%) | 21 (6.3%) |  |
| Female | 300 (94.6%) | 17 (5.4%) |  |
| **Child age** |  |  | .065 |
| 11-12 months old | 518 (94.9%) | 28 (5.1%) |  |
| 13-14 months old | 92 (90.2%) | 10 (9.8%) |  |
| **Birth older** |  |  | .686 |
| First child | 436 (94.4%) | 26 (5.6%) |  |
| Non-first child | 174 (93.5%) | 12 (6.5%) |  |
| **Primary caregiver** |  |  | .555 |
| Mother | 275 (93.5%) | 19 (6.5%) |  |
| Others | 335 (94.6%) | 19 (5.4%) |  |
| **Infant feeding practice** |  |  | .295 |
| Exclusive breastfeeding | 324 (95.6%) | 15 (4.4%) |  |
| Mixed feeding | 207 (92.4%) | 17 (7.6%) |  |
| Exclusive formula feeding | 79 (92.9%) | 6 (7.1%) |  |
| **Maternal education** |  |  | .400 |
| High school and below | 35 (94.6%) | 2 (5.4%) |  |
| College (2-3 years) | 94 (91.3%) | 9 (8.7%) |  |
| Bachelor's degree or higher | 481 (94.7%) | 27 (5.3%) |  |
| **Household income** |  |  | .456 |
| High | 151 (96.2%) | 6 (3.8%) |  |
| Middle | 429 (93.5%) | 30 (6.5%) |  |
| Low | 30 (93.8%) | 2 (6.2%) |  |
| Maternal oral health knowledge | 6.26 ± 1.64 | 6.03 ± 1.85 | .401 |
| Perceived susceptibility to ECC | 11.69 ± 1.80 | 11.71 ± 1.81 | .955 |
| Perceived severity to ECC | 14.23 ± 1.62 | 14.39 ± 1.17 | .562 |
| Perceived benefits of behavior | 28.81 ± 2.87 | 28.74 ± 2.45 | .870 |
| Perceived barriers of behavior | 21.91 ± 6.38 | 23.42 ± 5.80 | .157 |
| Self-efficacy | 13.68 ± 1.85 | 13.00 ± 2.13 | .030 |
| **Parental-assisting brushing** | |  | .808 |
| Yes | 521 (94.0%) | 33 (6.0%) |  |
| No | 89 (94.7%) | 5 (5.3%) |  |
| **Night feeding cessation** |  |  | .079 |
| Yes | 433 (93.1%) | 32 (6.9%) |  |
| No | 177 (96.7%) | 6 (3.3%) |  |
| **Sugar intake control** |  |  | .186 |
| Yes (≤2/d) | 474 (94.8%) | 26 (5.2%) |  |
| No (＞2/d) | 136 (91.9%) | 12 (8.1%) |  |
| **Dental visit in past 6 moths** | |  | .003 |
| Yes | 14 (77.8%) | 4 (22.2%) |  |
| No | 596 (94.6%) | 34 (5.4%) |  |
