# Supplementary material for: A Theory-Based Digital Intervention to Improve Maternal Oral Health Behaviors for Young Children: Quasi-Experimental Study
Source: JMIR Mhealth Uhealth. 2026 May 22;14:e79002. doi: 10.2196/79002 (PMC13197111; doi:10.2196/79002)
Supplement: Multimedia Appendix 5 [file mhealth-v14-e79002-s005.docx]

| **Multimedia Appendix 5. Intervention effects on behavioral outcomes: Based on complete-case analysis** | | | | | | |
| --- | --- | --- | --- | --- | --- | --- |
|  | Intervention group | Control group | Rate difference (RD) between group | | Odds ratio (OR) for intervention vs. control | |
|  |  |  | RD | *P*-value | OR | *P*-value |
| **Parental-assisting brushing** | |  |  |  |  |  |
| Baseline | 41/283 (14.5%) | 35/239 (14.6%) | -0.1% (-2.5, 2.3) | .940 | 1.00 |  |
| 6-month follow-up | 112/283 (39.6%) | 69/239 (28.9%) | 10.5% (2.5, 18.5) | .010 | 2.19 (1.06, 4.53) | .034 |
| 12-month follow-up | 116/283(41.0%) | 95/239 (39.7%) | 2.5% (-6.5, 11.5) | .592 | 1.18 (0.58, 2.42) | .651 |
| **Night feeding cessation** | |  |  |  |  |  |
| Baseline | 87/283 (30.7%) | 67/239 (28.0%) | 0.7% (-0.2, 1.7) | .137 | 1.00 |  |
| 6-month follow-up | 200/283 (70.7%) | 171/239 (71.5%) | -0.0% (-0.0, 0.0) | .790 | 1.21 (0.37, 4.13) | .623 |
| 12-month follow-up | 246/283 (86.9%) | 205/239 (85.8%) | 0.0% (0.0, 0.0) | .796 | 2.32 (0.55, 10.48) | .869 |
| **Sugar intake control** | |  |  |  |  |  |
| Baseline | 217/283 (76.7%) | 187/239 (78.2%) | -1.7% (-9.9, 6.4) | .676 | 1.00 |  |
| 6-month follow-up | 198/283 (70.0%) | 175/239 (73.2%) | -4.0% (-13.4, 5.5) | .409 | 0.91 (0.49, 1.71) | .772 |
| 12-month follow-up | 185/283 (65.4%) | 160/239 (66.9%) | -2.0% (-12.4, 8.4) | .707 | 1.02 (0.55, 1.89) | .952 |
| **Dental visit in past 6 moths** | |  |  |  |  |  |
| Baseline | 7/283 (2.5%) | 4/239 (1.7%) | 0.0% (-0.0, 0.1) | .652 | 1.00 |  |
| 6-month follow-up | 30/283 (10.6%) | 11/239 (4.6%) | 0.2% (-0.2, 0.6) | .360 | 1.94 (0.31, 12.21) | .480 |
| 12-month follow-up | 29/283 (10.2%) | 11/239 (4.6%) | 0.2% (-0.2, 0.6) | .372 | 1.81 (0.29, 11.38) | .529 |
