# Supplementary material for: A Theory-Based Digital Intervention to Improve Maternal Oral Health Behaviors for Young Children: Quasi-Experimental Study
Source: JMIR Mhealth Uhealth. 2026 May 22;14:e79002. doi: 10.2196/79002 (PMC13197111; doi:10.2196/79002)
Supplement: Multimedia Appendix 6 [file mhealth-v14-e79002-s006.docx]

| **Multimedia Appendix 6. Intervention effects on behavioral outcomes: Based on the last observation carried forward approach** | | | | | | |
| --- | --- | --- | --- | --- | --- | --- |
|  | Intervention group | Control group | Rate difference (RD) between group | | Odds ratio (OR) for intervention vs. control | |
|  |  |  | RD | *P*-value | OR | *P*-value |
| **Parental-assisting brushing** | |  |  |  |  |  |
| Baseline | 47/332 (14.2%) | 47/316 (14.9%) | -0.4% (-2.0, 1.2) | .673 | 1.00 |  |
| 6-month follow-up | 127/332 (28.3%) | 84/316 (26.6%) | 9.9% (3.0, 16.9) | .005 | 2.88 (1.43, 5.78) | .003 |
| 12-month follow-up | 132/332(39.8%) | 117/316 (37.0%) | 3.6% (-4.1, 11.2) | .361 | 1.46 (0.74, 2.91) | .277 |
| **Night feeding cessation** |  |  |  |  |  |  |
| Baseline | 104/332 (31.3%) | 79/316 (25.0%) | 2.2% (-3.3, 11.2) | .809 | 1.00 |  |
| 6-month follow-up | 229/332 (69.0%) | 216/316 (68.4%) | 1.3% (-4.2, 8.5) | .805 | 0.87 (0.29, 2.64) | .405 |
| 12-month follow-up | 279/332 (84.0%) | 266/316 (84.2%) | 0.1% (-0.3, 0.7) | .817 | 0.98 (0.25, 3.93) | .484 |
| **Sugar intake control** |  |  |  |  |  |  |
| Baseline | 259/332 (78.0%) | 241/316 (76.3%) | 2.8% (-4.5, 10.1) | .453 | 1.00 |  |
| 6-month follow-up | 230/332 (69.3%) | 228/316 (72.2%) | -2.9% (-11.6, 5.9) | .518 | 0.72 (0.40, 1.28) | .260 |
| 12-month follow-up | 217/332 (65.4%) | 210/316 (66.5%) | -0.7% (-10.4, 8.9) | .878 | 0.81 (0.46, 1.42) | .454 |
| **Dental visit in past 6 moths** |  |  |  |  |  |  |
| Baseline | 9/332 (2.7%) | 9/316 (2.8%) | -0.0% (-0.0, 0.0) | .706 | 1.00 |  |
| 6-month follow-up | 34/332 (10.2%) | 18/316 (5.7%) | 0.1% (-0.1, 0.2) | .356 | 2.96 (0.64, 13.68) | .164 |
| 12-month follow-up | 34/332 (10.2%) | 16/316 (5.1%) | 0.1% (-0.1, 0.2) | .300 | 3.85 (0.82, 17.95) | .086 |
