# Supplementary material for: A Theory-Based Digital Intervention to Improve Maternal Oral Health Behaviors for Young Children: Quasi-Experimental Study
Source: JMIR Mhealth Uhealth. 2026 May 22;14:e79002. doi: 10.2196/79002 (PMC13197111; doi:10.2196/79002)
Supplement: Multimedia Appendix 7 [file mhealth-v14-e79002-s007.docx]

| **Multimedia appendix 7. Intervention effects on toothbrushing with fluoridated toothpaste** | | | | | | |
| --- | --- | --- | --- | --- | --- | --- |
|  | Intervention group | Control group | Rate difference (RD) | | Odds ratio (OR) for intervention vs. control group | |
|  |  |  | RD | P value | OR | P value |
| Toothbrushing with fluoridated toothpaste | | | | | | |
| Baseline | 8/332 (2.4%) | 12/316 (3.8%) | -1.0% (-3.3, 1.2) | .372 | 1 |  |
| 12-month follow-up | 72/291(24.7%) | 53/260 (20.4%) | 4.~~5~~% (-3.4, 12.4) | .261 | 2.12 (0.78, 5.75) | .140 |
